# Postvaccine myocarditis and the use of antipyretics: Is there any relation?

**DOI:** 10.1002/clc.24061

**Published:** 2023-05-30

**Authors:** Maurish Fatima, Jawad Basit, Aleena Ahmed, Muhammad W. Murad, Sarya Swed, Huzaifa A. Cheema

**Affiliations:** ^1^ Department of Medicine King Edward Medical University Lahore Pakistan; ^2^ Department of Medicine Rawalpindi Medical University Rawalpindi Pakistan; ^3^ Department of Medicine Shanxi Medical University Shanxi China; ^4^ Faculty of Medicine Aleppo University Aleppo Syria

To the Editor,

We have read the correspondence by Stephen A. Hoption Cann regarding antipyretics and vaccine‐induced myocarditis with keen interest and are grateful to the colleague for the insightful comments. The author has highlighted the similar side effects of both the smallpox vaccine (after the first dose of ACAM2000 and Dryvax) and the coronavirus disease 2019 (COVID‐19) vaccine (after the second dose of messenger RNA [mRNA] vaccine); the subsequent treatment with a high dosage of antipyretics; and has also commented on the possible role of frequent use of antipyretics in triggering postvaccine myocarditis.^1^

While we do agree that both smallpox and mRNA vaccines share the feature of myocarditis being more common, however, the second dose of mRNA vaccine is associated with greater severity of side effects such as headaches, myalgias, fever, and chest pain. This, in turn, leads to a frequent and increased use of antipyretics, generally ibuprofen and acetaminophen, suggesting a possible role of antipyretics in the development of this condition^2^ It is also important to remember that the timing of antipyretic administration in connection to the beginning of myocarditis symptoms should also be taken into account while examining their potential significance in the development of this condition following the COVID‐19 vaccine.^3^ Although the development of Reye syndrome following the widespread use of aspirin in viral infections and the research using animal models have suggested that nonsteroidal anti‐inflammatory drugs (NSAIDs), primarily used early in the course of viral myocarditis, may aggravate the condition and raise mortality, however, there is not much evidence available to support this hypothesis from experimental studies and randomized controlled trials.^4^, ^5^ We do agree with the insightful comments by the author; however, the lack of substantial evidence and research makes it difficult to devise a cause–effect association.^3^, ^6^

Among the several proposed mechanisms regarding vaccine‐induced myocarditis that have been discussed in the literature, one of the mechanisms is that the vaccine triggers an autoimmune response in susceptible individuals where antibodies to severe acute respiratory syndrome coronavirus 2 spike glycoproteins crossreact with myocardial contractile proteins, leading to inflammation of the heart muscle.^7^ Another proposed mechanism is vaccine‐induced hyperimmunity, that is, the production of proinflammatory cytokines, which can cause damage to the heart tissue. However, it is important to note that the exact mechanisms underlying vaccine‐induced myocarditis are not fully understood, and further research is needed to better understand this condition's pathophysiology.^8^

Additionally, our review focused on incidence, clinical presentation, management, and any association of myocarditis and pericarditis with the COVID‐19 vaccines in children and adolescents by incorporating the findings from case reports, case series, and observational studies. In our review, six observational studies and seven case series and case reports were included, where the latter comprised only 0.000016% of the total patient population and very limited information was available regarding the use of NSAIDs and other antipyretics for the treatment of disease. Based on the available data, it is difficult to comment on the association of postvaccine myocarditis with the use of antipyretics.^2^

To identify whether antipyretics may play a part in myocarditis caused by vaccines, there is a need for more research. This will allow us to provide recommendations for their use after immunization based on scientific data and guarantee the security of COVID‐19 vaccine recipients.
